# Cut-off value for interleukin-34 as an additional potential inflammatory biomarker for estimation of slow coronary flow risk

**DOI:** 10.1186/s12872-023-03677-y

**Published:** 2024-01-02

**Authors:** Mehdi Karasu, Hasan Ata Bolayır

**Affiliations:** 1 Department of Cardiology, Fethi Sekin Sehir Hastanesi, Elazıg, Turkey; 2 Department of Cardiology, Malatya Turgut Özal Üniversitesi Kardiyoloji ABD, Malatya, Turkey

**Keywords:** IL-34, hsCRP, Slow coronary flow (SCF)

## Abstract

**Background:**

Inflammatory markers may provide insights into the underlying mechanisms of slow coronary flow (SCF), including subclinical atherosclerosis and endothelial dysfunction. Interleukin-34 (IL-34), known for its role in immuno-inflammatory diseases, might hold significance in SCF. We aimed to explore the potential association between IL-34 and SCF in patients undergoing diagnostic elective coronary angiography.

**Methods:**

This observational, cross-sectional study enrolled 256 participants: 124 with SCF and 132 with normal coronary flow (NCF). All participants had undergone outpatient coronary angiography for suspected coronary artery disease. SCF assessment employed the TIMI frame count (TFC) for quantifying coronary flow rate.

**Results:**

SCF patients exhibited significantly elevated TFC in all three major coronary arteries compared to controls (*p* < 0.05). IL-34 displayed a noteworthy positive correlation with average TFC [for all participants: r = 0.514, *p* < 0.001; for SCF patients: r = 0.526, *p* < 0.001; for normal controls: r = -0.288, *p* > 0.05]. Similarly, high-sensitivity C-reactive protein (hsCRP) showed a significant and positive relationship with average TFC [for all participants: r = 0.504, *p* < 0.001; for SCF patients: r = 0.558, *p* < 0.001; for normal controls: r = -0.148, *p* > 0.05]. SCF patients presented coronary arteries of larger size compared to controls.

**Conclusion:**

Mean coronary diameter and IL-34 emerged as independent predictors of SCF. Additionally, hsCRP, mean coronary diameter, and IL-34 exhibited a positive correlation with mean TFC values. IL-34 appears to be a more effective indicator than hsCRP in SCF patients.

## Introduction

Slow coronary flow (SCF), initially defined by Tambe et al. through visual assessment of angiographic images, refers to the delayed perfusion of distal vessels despite the absence of organic heart disease and overt coronary artery stenosis. SCF can manifest in a single vessel or involve multiple vessels simultaneously [1–3]. It is reported in the literature that approximately 7% of patients undergoing coronary angiography exhibit SCF [4]. Notably, SCF holds clinical significance beyond manifesting as chest pain, as it can progress to complications such as acute coronary syndrome, fatal arrhythmias, and sudden death [1, 5–8].

Despite numerous studies investigating potential underlying causes—ranging from subclinical atherosclerosis and endothelial damage to microvascular dysfunction, inflammation, and abnormal blood cell distribution—the etiopathogenesis of SCF remains incompletely understood [2, 9].

Inflammatory markers’ aberrations might shed light on mechanisms contributing to SCF, including subclinical atherosclerosis and endothelial dysfunction. Inflammation plays a pivotal role across all stages of atherosclerosis [10]. Components like high-sensitivity C-reactive protein (hsCRP), albumin, uric acid, endothelial damage-associated adhesion molecules, and peripheral blood cells actively involved in inflammation have shown associations with SCF [11–14].

Interleukin-34 (IL-34), originally linked to macrophage colony-stimulating factor (M-CSF), exerts influence on the inflammatory process by modulating monocyte and macrophage life cycles [15, 16]. Notably, IL-34 plays crucial roles in various immuno-inflammatory diseases, including Sjögren’s syndrome, rheumatoid arthritis, and inflammatory bowel diseases [17–19].

Increased IL-34 levels have been significantly linked to ischemic cardiomyopathy and may serve as an indicator for cardiovascular death, decompensation-related hospitalization, and all-cause mortality in heart failure patients [20, 21]. Elevated IL-34 levels also correlate with heightened heart failure presence and severity in acute myocardial infarction patients, along with increased cardiovascular mortality [22]. Despite these insights, the relationship between IL-34 and SCF remains unclarified. Consequently, our study aims to explore the potential association between IL-34 and SCF in patients undergoing diagnostic elective coronary angiography.

## Methods

The methodology employed in this research was observational and cross-sectional in nature. A total of 256 participants were involved, comprising 124 individuals with SCF (slow coronary flow) and 132 controls with NCF (normal coronary flow). These participants had undergone outpatient coronary angiography between January 2022 and May 2023 at our facilities due to suspected coronary artery disease (CAD). All participants exhibited normal coronary arteries as confirmed by angiography, varying in their coronary flow rates, and none displayed atherosclerotic lesions.

All individuals included in the study experienced chest pain or symptoms suggestive of angina, verified through myocardial perfusion studies or treadmill tests. They met the criteria for cardiac syndrome-X, demonstrating: [1] angina primarily triggered by exertion, suggestive of CAD; [2] instances of randomly occurring or induced angina associated with anomalies in coronary blood flow or myocardial ischemia; [3] angiographically normal coronary arteries; [4] absence of other definitive cardiac diseases. Each patient underwent a comprehensive physical examination by a cardiology physician, their medical history was documented, and cardiac catheterization was performed during a clinic visit, with data recorded and filed in the hospital’s coronary angiography laboratory.

Exclusion criteria encompassed pre-existing diseases (e.g., peripheral vascular or coronary), ectatic coronary arteries, non-ischemic dilated cardiomyopathy, renal or hepatic dysfunctions, ongoing inflammation or infection, hematological abnormalities, and malignancies. Importantly, none of the participants were under vasoactive prescription drugs during the study period.

All patients were informed about the aims of the study and their Written/verbal informed consent for participation was obtained. This study was approved by Fırat University Ethics Committee(06.2022-8744) in accordance with the International Code of Ethics and the Declaration of Helsinki.

### Evaluating biochemical parameters

Blood was collected from each patient’s femoral artery at the time of femoral puncture, just before coronary angiography, following a fasting period of 12 h. After centrifugation at 4000 × g for 10 min, serum was separated and stored at -80°C until required. Complete blood count and differentials were analyzed from the collected blood using an automated analyzer. High sensitivity C-reactive protein (hsCRP), total cholesterol, triglycerides, creatinine, low-density lipoprotein (LDL) cholesterol, and high-density lipoprotein (HDL) cholesterol were measured using an automated analyzer. Additionally, plasma IL-34 levels were assessed using ELISA.

Coronary angiography was performed using the Judkins method through the femoral approach, with iopromide as the contrast medium. The study participants underwent coronary flow assessment for SCF using the Thrombolysis In Myocardial Infarction (TIMI) frame count (TFC) method. Two independent clinicians, blinded to clinical data, quantified coronary flow using TFC. The TFC involved determining the number of cine frames required for contrast to reach specific distal coronary landmarks in the left circumflex (LCX), left anterior descending (LAD), and right coronary arteries (RCA). Normal TFC averages are reported as 22.2 ± 4.1 frames for LCX, 36.2 ± 2.6 frames for LAD, and 20.4 ± 3 frames for RCA. Corrected TFC (cTFC) for LAD was calculated by dividing by 1.7, resulting in 21.1 ± 1.5 frames. SCF diagnosis was based on cTFC exceeding 2 standard deviations from the reported range for each vessel. Vessel lengths and ostial diameters were measured using quantitative coronary angiography (QCA).

### Statistics

The statistical analysis was performed using SPSS software (version 20.0 for Windows). Continuous variables were represented as means with standard deviations, while categorical variables were depicted as percentages and evaluated using the Chi-square test. The normality of data distribution was assessed through the Kolmogorov-Smirnov test.

For normally distributed continuous variables, a two-sample T-test was employed in the univariate analysis. Non-normally distributed variables underwent analysis using the Mann-Whitney U test. Logistic regression analysis was utilized to identify independent risk factors associated with SCF.

Throughout the analysis, significance was determined at a threshold of *p* < 0.05 for all statistical tests conducted.

## Results

Table 1 in the study outlines the crucial clinical parameters of all participants. Notably, there were no significant variations observed between the SCF and control groups concerning gender, age, presence of hypertension, history of smoking, or diabetes incidence (*p* > 0.05). Furthermore, there were no disparities detected in lipid parameters or fasting glucose levels between the two groups (*p* > 0.05).

**Table 1:** Demographic and clinical characteristics of study participants

Variables	SCF (n = 124)	NCF (n = 132)	*p
Age, years	52 ± 10	54 ± 13	0.54
Male gender, n	59 (%49)	66 (%50)	0.82
BMI, kg/m2	30.2 ± 8.1	31.7 ± 4.2	0.66
Hypertension, n	37 (%30)	42 (%32)	0.86
Diabetes mellitus, n	50 (%40)	50 (%38)	0.77
Hyperlipidemia, n	27 (%22)	32 (%24)	0.68
Cigarette smoking, n	37 (%30)	35 (%27)	0.24
Family history of CAD, n	74 (%60)	77 (%58)	0.82
Fasting glucose, mg/Dl	111 ± 34	101 ± 28	0.54
Total cholesterol, mg/dL	198 ± 42	192 ± 33	0.68
Triglycerides, mg/dL	154 ± 76	148 ± 58	0.18
HDL cholesterol, mg/dL	40 ± 12	44 ± 14	0.12
LDL cholesterol, mg/dL	168 ± 33	144 ± 28	0.16
*hsCRP, mg/dL*	*3.8 ± 0.4*	*1.1 ± 0.2*	*0.008*
*IL-34, pg/mL*	*38.8 ± 4.4*	*10.3 ± 4.2*	*< 0.001*
***TIMI frame count measurements***			
*LAD*	*62 ± 26*	*28 ± 5*	*< 0.001*
*LAD(corrected)*	*37 ± 16*	*16 ± 2*	*< 0.001*
*LCx*	*28 ± 10*	*19 ± 4*	*< 0.001*
*RCA*	*40 ± 22*	*18 ± 7*	*< 0.001*
*Mean*	*37 ± 11*	*18 ± 5*	*< 0.001*
***The length of epicardial coronary arteries***			
LAD,mm	168 ± 21	171 ± 18	0.86
LCx,mm	124 ± 32	121 ± 26	0.74
RCA,mm	180 ± 37	168 ± 33	0.44
***Diameters of coronary arteries***			
*LAD,mm*	*3.86 ± 0.46*	*3.48 ± 0.52*	*0.022*
*LCx,mm*	*3.52 ± 0.62*	*3.24 ± 0.72*	*0.046*
*RCA,mm*	*3.66 ± 0.62*	*3.12 ± 0.56*	*0.009*
***Coronary flow velocities***			
*LAD,mm/s*	*101.7 ± 44.0*	*184.6 ± 57.8*	*< 0.001*
*LCx,mm/s*	*150.9 ± 57.6*	*201.7 ± 61.2*	*< 0.001*
*RCA,mm/s*	*167.6 ± 70.9*	*279.1 ± 76.4*	*< 0.001*

The data are displayed in three formats: mean ± SD, median (interquartile range), and as numbers with percentages. Statistical analysis was performed using Student’s t-test, Mann-Whitney U test, and Chi-square test as appropriate. The abbreviations used are as follows: BMI (body mass index), HDL (high-density lipoprotein), LAD (left anterior descending artery), LCx (left circumflex artery), LDL (low-density lipoprotein), NCF (normal coronary flow), NS (not significant), RCA (right coronary artery), SCF (slow coronary flow), hsCRP (high sensitivity C-reactive protein), and IL-34 (interleukin-34).

### TFC

The patients diagnosed with SCF exhibited significantly elevated TFC levels across all three major coronary arteries compared to the control group (*p* < 0.05). Interestingly, vessel lengths did not differ between the SCF and control groups. However, the SCF patients displayed significantly larger coronary artery sizes, whereas the control group showcased notably higher coronary flow rates (*p* < 0.05).

**Fig. 1:**
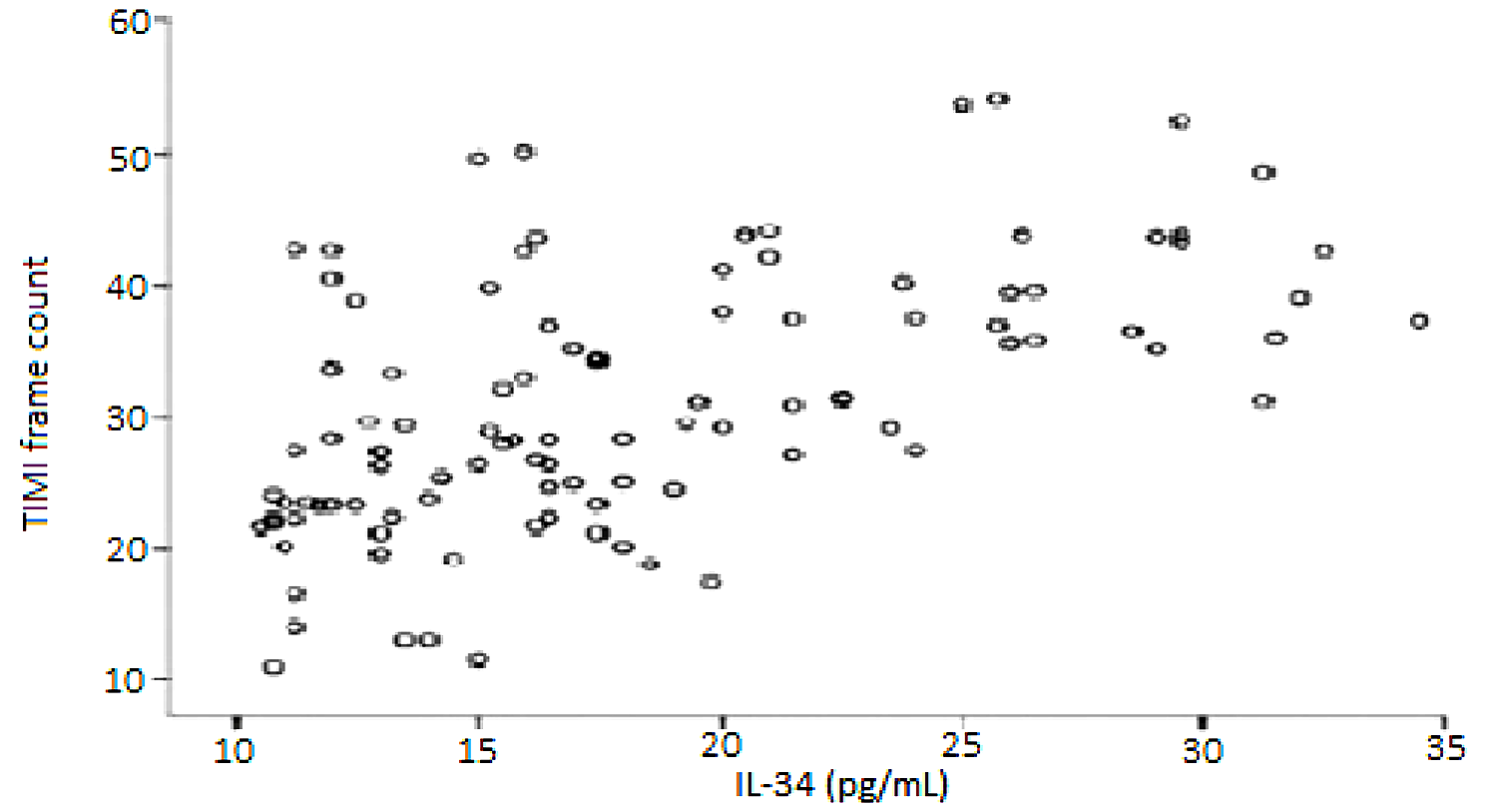
The correlation of IL-34 levels with TIMI frame count in patients with SCF.

Notably, IL-34 demonstrated a significant positive correlation with average TFC levels: among all participants (r = 0.514, *p* < 0.001), within the SCF patients (r = 0.526, *p* < 0.001), and for normal controls (r = -0.288, *p* > 0.05) (refer to Fig. 1). Additionally, hsCRP also exhibited a significant positive association with average TFC levels: across all participants (r = 0.504, *p* < 0.001), within SCF patients (r = 0.558, *p* < 0.001), and for normal controls (r = -0.148, *p* > 0.05).

Individuals diagnosed with SCF showed significantly higher average serum levels of hsCRP and IL-34 compared to the control group (hsCRP: 3.8 ± 0.4 vs. 1.1 ± 0.2 mg/dL, *p* = 0.008; IL-34: 38.8 ± 4.4 vs. 10.3 ± 4.2 pg/mL, *p* < 0.001, respectively). Through logistic regression analysis (as displayed in Table 2), it was evident that the average coronary diameter was a robust predictor of SCF (OR: 7.364, 95% CI: 1.988–28.64, *p* = 0.004). Conversely, IL-34 demonstrated a weaker association with SCF prediction (OR: 1.044, 95% CI: 1.006–1.084, *p* = 0.018).

**Table 2:** The independent relationship of IL-34 with slow coronary flow phenomenon

Variables	Slow coronary flow (Dependent variable)	
	OR (95%CI)	*p
IL-34, pg/mL	1.044 (1.006–1.084)	0.018
Mean coronary diameter	7.364 (1.988–28.64)	0.004

**Fig. 2:**
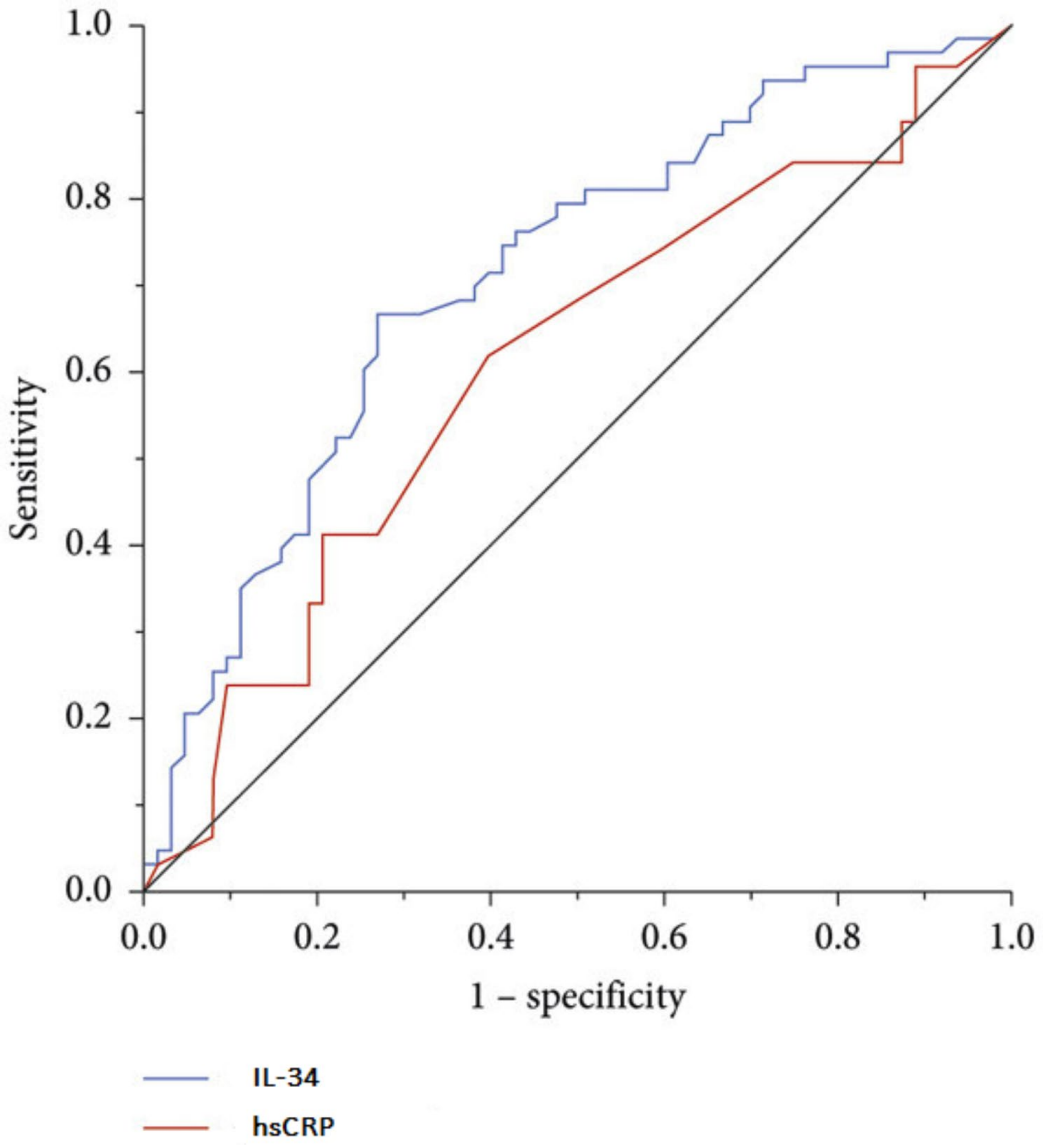
ROC curve analyses of the predictive power of plasma IL-34 and hs-CRP levels

The utilization of a receiver operating characteristic (ROC) curve allowed us to evaluate the sensitivity and specificity of IL-34 and hsCRP in detecting SCF within the study participants. The results presented in Fig. 2 highlighted that plasma IL-34 levels exhibited a significant predictive capacity in distinguishing individuals with SCF from those with NCF (AUC = 0.804, 95% CI: 0.735–0.872, *p* < 0.001). Notably, this predictive ability surpassed that of plasma hsCRP (AUC = 0.617, 95% CI: 0.533–0.702, *p* = 0.010).

Moreover, our findings suggest that a cutoff value of 29.95 pg/mL for plasma IL-34 could effectively differentiate individuals with SCF from those with NCF, demonstrating a specificity of 74.2% and a sensitivity of 90.7%.

## Discussion

To the best of our knowledge, this is the first study to establish an association between interleukin-34 (IL-34) and slow coronary flow (SCF). Our investigation revealed that mean coronary diameter and IL-34 emerged as independent predictors of SCF. Furthermore, we observed a positive correlation between high-sensitivity C-reactive protein (hsCRP), mean coronary diameter, IL-34, and mean TIMI frame count (TFC) values. Notably, IL-34 exhibited superior predictive potential compared to hsCRP in patients with SCF.

In contrast to the commonly used TIMI flow velocity assessment [23], the corrected TIMI frame count (TFC) [24] offers an objective measure that standardizes flow evaluation. As such, we adopted TFC in o ur study.

Microvascular degeneration, implicated in SCF etiology, is characterized by myointimal thickening and endothelial edema [3, 25]. The endothelium plays a pivotal role in regulating vascular tone, platelet activity, leukocyte adhesion, and vascular smooth muscle proliferation. Endothelial dysfunction’s role in SCF is underscored by Sezgin AT et al.‘s findings of reduced endothelium-dependent brachial artery dilation in SCF patients [26]. Cin et al. [27] demonstrated diffuse intimal thickening, calcification, and non-obstructive atheromatous changes along coronary vessel walls in SCF patients. A study by Aşkın et al. examined the carotid flow rate in patients with slow coronary flow, demonstrating that endothelial dysfunction, microvascular resistance, and small vessel disease similarly influenced both carotid flow rate and slow coronary flow [28]. Another study conducted by Aşkın demonstrated an association between coronary slow flow and the heart rate recovery index, known as a predictor of cardiovascular diseases [29].

Recent years have seen the frequent use of peripheral blood cells and related indices to explore the inflammation-cardiovascular disease nexus. Neutrophil-lymphocyte ratio, platelet-lymphocyte ratio, and systemic immune-inflammatory index have linked to coronary artery disease, acute coronary syndromes, heart failure, valve diseases, hypertension, and SCF [30–33]. SCF’s association with adhesion molecules like intercellular adhesion molecule-1, vascular cell adhesion molecule-1, and E-selectin is documented [13]. Other markers such as erythrocyte distribution width and serum uric acid levels also correlate with SCF [34, 35]. Li et al. [36] revealed elevated plasma high-sensitivity C-reactive protein and interleukin-6 concentrations in SCF patients.

IL-34, a proinflammatory cytokine, stimulates chemokines and cytokines like monocyte chemoattractant protein, IL-6, and IL-8 [16]. It plays roles in inflammatory cell differentiation and migration, including macrophages and monocytes [37]. Preisser L et al. linked IL-34 to profibrotic macrophages, releasing transforming growth factor β, platelet-derived growth factor, and galectin-3—factors impacting heart failure development [38]. Xi R et al. [21] demonstrated significantly increased serum IL-34 in ischemic cardiomyopathy, correlating with ischemic heart failure presence and severity. Fan Q et al. [39] associated IL-34 with coronary artery disease presence and severity. Li Z et al. [40] noted elevated IL-34 in CAD patients, correlating positively with hs-CRP levels. Zorena K et al. [41] underscored IL-34’s enhanced discrimination over C-reactive protein for vascular diabetes complications.

Similarly, our findings revealed elevated IL-34 levels in SCF patients compared to those with normal coronary flow, accompanied by greater mean coronary diameter. Both IL-34 and mean coronary diameter independently predicted SCF in our study. Moreover, hsCRP exhibited a significant positive correlation with average TFC. ROC curve analysis highlighted IL-34’s potential superiority to hsCRP as an indicator in SCF patients.(AUC = 0.804 vs. AUC = 0.617).

### Study limitations

Several limitations are associated with the present study. Primarily, the study’s single-center, cross-sectional, and observational nature, coupled with a relatively modest sample size, may limit the generalizability of the findings. The diagnosis of slow coronary flow (SCF) relied solely on angiographic observations, potentially omitting additional diagnostic nuances. Furthermore, our study did not investigate the potential normalization of SCF following dipyridamole or nitroglycerin infusion. An absence of follow-up prevented the assessment of clinical outcomes within the study population. As a result, larger-scale prospective randomized controlled trials are imperative to substantiate the relationship between interleukin-34 (IL-34) and SCF.

## Conclusion

Elevated levels of IL-34 signal the involvement of inflammation, microvascular dysfunction, subclinical atherosclerosis, and endothelial damage in the pathogenesis of slow coronary flow (SCF). Moreover, heightened IL-34 levels could potentially serve as an early indicator of compromised coronary blood flow.

## Data Availability

All needed data can be obtained from corresponding author.
